# TNFα-Mediated Liver Destruction by Kupffer Cells and Ly6C^hi^ Monocytes during *Entamoeba histolytica* Infection

**DOI:** 10.1371/journal.ppat.1003096

**Published:** 2013-01-03

**Authors:** Elena Helk, Hannah Bernin, Thomas Ernst, Harald Ittrich, Thomas Jacobs, Joerg Heeren, Frank Tacke, Egbert Tannich, Hannelore Lotter

**Affiliations:** 1 Bernhard Nocht Institute for Tropical Medicine, Hamburg, Germany; 2 Department and Clinic for Diagnostic and Interventional Radiology, University Medical Center Hamburg-Eppendorf, Hamburg, Germany; 3 Department of Biochemistry and Molecular Cell Biology, University Medical Center Hamburg-Eppendorf, Hamburg, Germany; 4 Department of Medicine III, University Hospital Aachen, Aachen, Germany; University of California, San Diego, United States of America

## Abstract

Amebic liver abscess (ALA) is a focal destruction of liver tissue due to infection by the protozoan parasite *Entamoeba histolytica* (*E. histolytica*). Host tissue damage is attributed mainly to parasite pathogenicity factors, but massive early accumulation of mononuclear cells, including neutrophils, inflammatory monocytes and macrophages, at the site of infection raises the question of whether these cells also contribute to tissue damage. Using highly selective depletion strategies and cell-specific knockout mice, the relative contribution of innate immune cell populations to liver destruction during amebic infection was investigated. Neutrophils were not required for amebic infection nor did they appear to be substantially involved in tissue damage. In contrast, Kupffer cells and inflammatory monocytes contributed substantially to liver destruction during ALA, and tissue damage was mediated primarily by TNFα. These data indicate that besides direct antiparasitic drugs, modulating innate immune responses may potentially be beneficial in limiting ALA pathogenesis.

## Author Summary

Amebic liver abscess (ALA), an infectious disease caused by the protozoan parasite *Entamoeba histolytica* is characterized by severe focal liver damages. According to its name giving activity, destruction of liver tissue by *E. histolytica* has been attributed to parasite-specific effector molecules. However, abscess lesions contain considerable numbers of innate immune cells, such as neutrophils and macrophages, raising the question whether these host cells might contribute to the disease as well. We have investigated the role of the host immune response during ALA development using a mouse model for the disease. The results indicated that despite the presence of considerable numbers of neutrophils within the abscess lesions, these cells were dispensable for both the control of the disease and the tissue damage. On the other hand, two subsets of immune cells, the liver resident Kupffer cells and the inflammatory Ly6C-expressing monocytes were identified as the main effector cells responsible for liver tissue destruction. Furthermore, TNFα produced by the Ly6C-expressing monocytes, was found to be a cytokine that is critically involved in abscess development. Thus, our finding that host immune mechanisms are indeed responsible for liver tissue destruction during ALA development may change the view on the pathological mechanism of amebic disease.

## Introduction

*Entamoeba histolytica (E. histolytica)* is a protozoan parasite that colonizes the human gut. Infection is typically asymptomatic; however, in about 10% of cases, *E. histolytica* trophozoites penetrate into the gut tissue and cause hemorrhagic colitis or spread to the liver and induce amebic liver abscesses (ALA), a progressive focal destruction of liver tissue. Invasive amebiasis is estimated to constitute approximately 50 million cases annually worldwide [1].

Over the past several decades, most studies of ALA focused on parasite-specific pathogenicity factors such as the D-galactosamine-inhibitable (Gal/GalNAc) adherence lectin, the pore forming peptides (amebapores), and cysteine peptidases, as causative agents in the penetration of host tissue and induction of invasive disease [2]–[4]. However, homologues of a majority of the genes that are assumed to be essential for pathogenicity are also present in the non-pathogenic species, *E. dispar*, which is genetically very closely related to *E. histolytica* but does not cause clinical symptoms [5].

Beside parasite-specific effector molecules, there is accumulating evidence that host-mediated mechanisms also contribute to disease progression in the liver. For example, adult males are more susceptible to ALA, despite the fact that infection with *E. histolytica* is more prevalent in women and children [6]. In addition, histological analysis of liver sections from human ALA patients, as well as from ALA rodent models, consistently shows massive accumulation of inflammatory cells, primarily neutrophils, and macrophages, within the abscess [7]–[9]. While these immune cells represent the first line of defense against microorganisms, such an overwhelming immune response and the antimicrobial factors released by inflammatory cells could damage the host tissues as well [10], [11].

Neutrophils are terminally differentiated cells characterized by surface expression of Ly6G [12]. They are rapidly recruited to sites of injury or infection, where they generate and release reactive oxygen intermediates (ROI) and proteolytic enzymes directed at killing and phagocytosis of pathogens [13]. Subsequently, neutrophils undergo cell death, which potentially increases the amount of cytotoxic molecules at the site of infection [10].

Resident macrophages in the liver, termed Kupffer cells, also contribute to host antimicrobial defenses. However, in animal models of hepatotoxic liver injury, Kupffer cells also exhibit tissue-destructive potential [14]. Recent reports suggest that there are two subpopulations of Kupffer cells that can be differentiated by phenotype and function [15]. All Kupffer cells express the macrophage-restricted glycoprotein F4/80 [16]; however, subsets can be further characterized by the expression of CD11b, a C3b receptor present on the surface of monocytes and macrophages [17], or CD68, also known as macrosialin [18]. CD11b^+^ cells mainly produce cytokines and show weak cytolytic activity. By contrast, CD68^+^ cells exhibit phagocytic and cytotoxic activity via production of reactive oxygen species [19] and superoxide [20].

A heterogeneous CD11b^+^ monocyte population has been identified that expresses C-C chemokine receptor 2 (CCR2) and also shows high-level cell surface expression of Ly6C (Ly6C^hi^CCR2^+^). Secretion of C-C chemokine ligand 2 (CCL2) by injured or inflamed tissue cells induces migration of these Ly6C^hi^CCR2^+^ monocytes from the bone marrow to the site of infection, where they are involved in the immune defense responses against pathogenic microorganisms [21]. Activated Ly6C^hi^CCR2^+^ monocytes exhibit strong antimicrobial activity and promote pro-inflammatory immune responses [22]. In particular, in the liver, Ly6C^hi^CCR2^+^ monocytes give rise to TNFα- and iNOS-producing dendritic cells (TipDCs), inflammatory macrophages, and inflammatory DCs [22]. A number of models of hepatotoxicity show that CCR2^−/−^ knockout mice are protected from liver injury, indicating the tissue destructive potential of Ly6C^hi^CCR2^+^ inflammatory monocytes [23]–[26].

The aim of the present study was to investigate the contribution of neutrophils, resident Kupffer cells, and Ly6C^hi^ monocytes to liver injury in ALA using an immune competent mouse model for ALA [9]. The recruitment dynamics of these three immune cell subsets was investigated by immunohistochemistry and flow cytometry. The effects of selective cell depletion and neutralization on abscess development were monitored by magnetic resonance imaging (MRI). Here we showed for the first time that not parasite-derived hepatotoxic substances but TNFα released by Kupffer cells and Ly6C^hi^ monocytes is critical for tissue damaging effects during ALA development.

## Results

### Localization of neutrophil granulocytes (neutrophils) and macrophages in liver tissue during ALA formation

**Figure 1:**
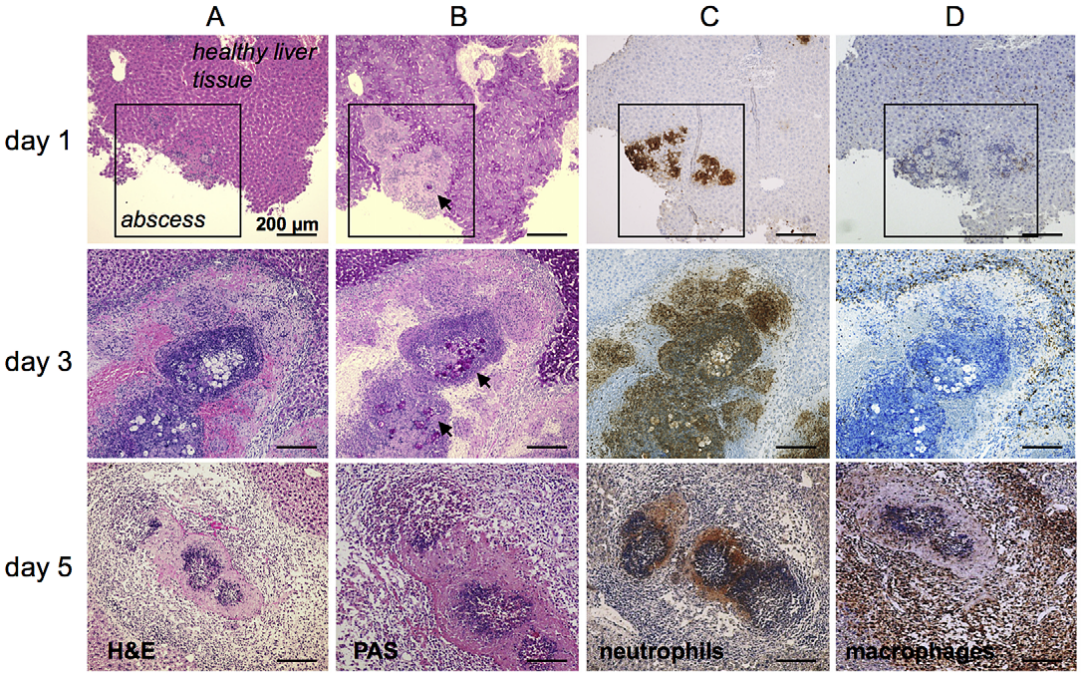
Histological and immunohistochemical characterization of cell infiltrates during ALA. (A) H&E staining of mouse liver abscesses (indicated by the square in the top row of images) at the indicated times post-infection with *E. histolytica* trophozoites. (B) PAS staining shows *E. histolytica* trophozoites (arrowheads) within the abscess. (C and D) Tissue sections were stained with anti-7/4 (C) and anti-F4/80 (D) antibodies followed by HRP-conjugated secondary antibody to detect neutrophils and macrophages, respectively (brown).

To determine the sites of neutrophil and liver macrophage infiltration within the liver abscess during ALA development, liver tissue sections were obtained over several days following infection with *E. histolytica* and analyzed by histology. Hematoxylin & Eosin (H&E) and periodic acid-Schiff (PAS) staining of paraffin-embedded samples was used to visualize host cells and amebic trophozoites, respectively, within the abscess lesion (Figure 1). Discrete infiltrates of predominately mononuclear cells (Figure 1A, Day 1) that co-localized with amebic trophozoites (Figure 1B, Day 1) were evident on Day 1 post-infection. Immunohistochemical staining demonstrated that most of the infiltrated cells were neutrophils (Figure 1C, Day 1), with a few macrophages (Figure 1D, Day 1). By Day 3 post-infection, the cellular infiltrate in the abscess had increased and become more organized (Figure 1A, Day 3). Mononuclear cells were located within the center of the abscess surrounding the trophozoites and at the periphery (Figure 1A and B, Day 3). Mononuclear cells within the abscess were identified as neutrophils (Figure 1C, Day 3), while macrophages accumulated predominantly at the boundary of the abscess (Figure 1D, Day 3). By Day 5 post-infection, H&E staining revealed a denser, but reduced, cellular infiltrate in the center of the abscess (Figure 1A, Day 5). The immune cell infiltrate was no longer dominated by neutrophils (Figure 1C, Day 5), but by F4/80-positive macrophages (Figure 1D, day 5). These results clearly demonstrate the differential migration and localization of neutrophils and macrophages during abscess formation relative to the location of amebic trophozoites within the liver tissue, which might suggest distinct functions of these myeloid immune cells for ALA-related hepatopathogenesis.

### The role of neutrophils in ALA development

**Figure 2:**
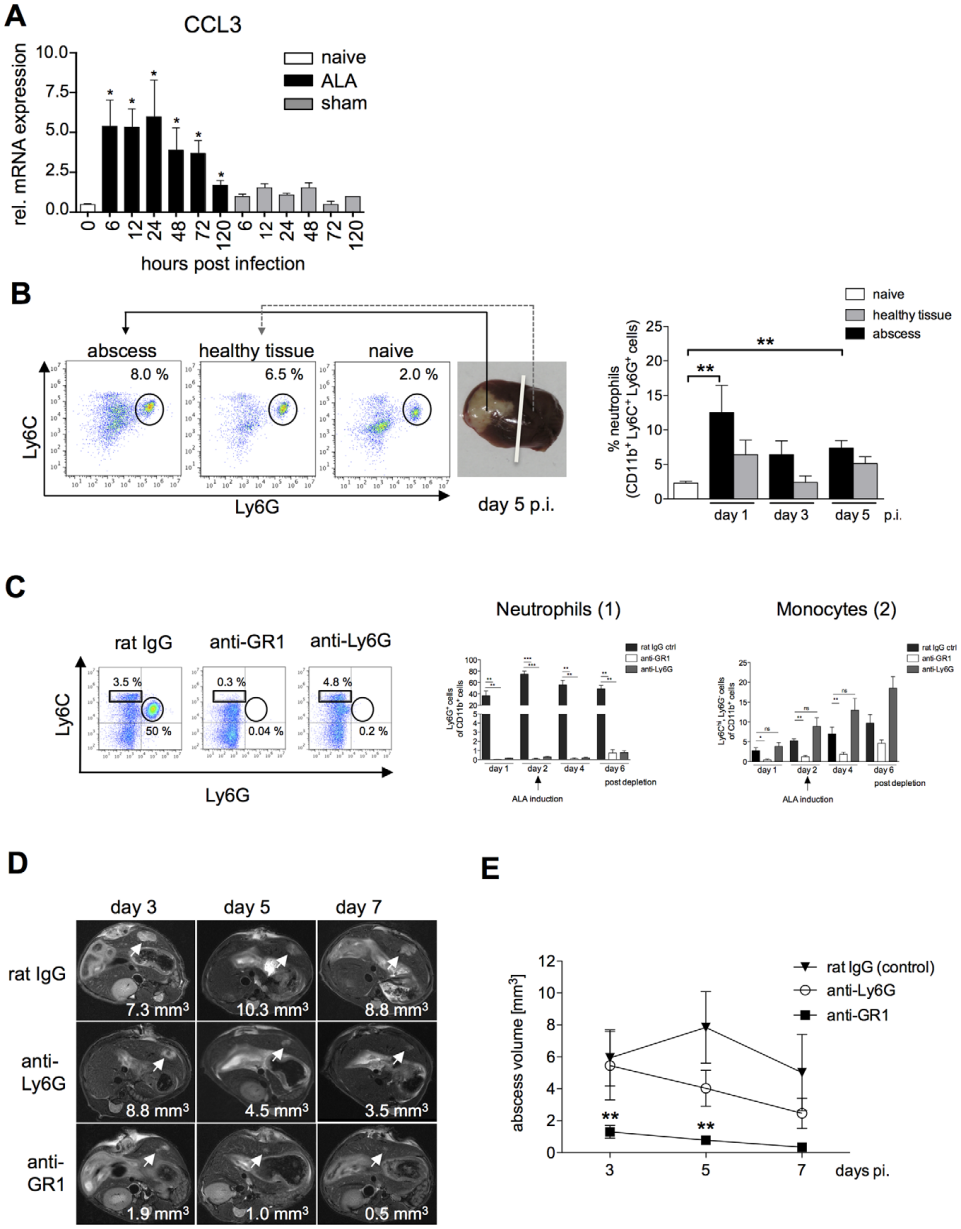
Neutrophil recruitment during ALA and effects of selective neutrophil depletion. (A) Levels of CCL3 mRNA were increased in liver tissue of infected mice (ALA) compared with sham-operated mice (sham) or naïve mice (naïve). (B) Gating strategy to define neutrophils isolated from the abscessed region of the infected liver (abscess), a healthy region from the same liver lobe (healthy tissue), and liver tissue of a naïve mouse (naïve) following intrahepatic amebic infection (n = 3–4 animals/group). Neutrophils were defined as CD11b^+^Ly6G^+^ cells. (C) FACS analysis of blood leukocytes at the indicated time points after neutrophil depletion with anti-Ly6G and anti-GR1 antibodies; control mice were subjected to depletion with a non-specific immunoglobulin (rat IgG). CD11b pre-gated cells were further defined as neutrophils by the expression of Ly6G (n = 5 animals/group) and as blood monocytes by the expression of Ly6C (n = 5 animals/group). Depletion efficacy was estimated on indicated time points after the first treatment. (D) Representative T_2_ weighted MRI images of mouse liver tissue showing the size of the abscess (arrowheads) following depletion with anti-Ly6G or anti-GR1 antibodies compared to control mice at the indicated times post-infection. (E) Abscess volume in control mice and anti-Ly6G- and anti-GR1-treated mice. Data represent the mean ± SEM of three independent experiments (n = 9–13); *P*-values were determined by the unpaired Student's t-test (^*^ *P*<0.05).

Since neutrophils appeared to be the predominant cell type in the immune cell infiltrates during the first three days after infection, we analyzed total RNA isolated from abscessed liver tissue by quantitative real-time-PCR (qPCR) to determine the mRNA expression kinetics of C-C chemokine ligand 3 (CCL3), also known as macrophage inflammatory protein-1α (MIP-1α), a chemokine that participates in the recruitment of neutrophils. As shown in Figure 2A, CCL3 expression was already elevated by 6 h post-infection compared with that in uninfected (naïve) liver tissue (*P*<0.03). CCL3 mRNA expression was also slightly increased in liver tissue from sham-operated animals that were injected with amebic culture medium alone.

To further quantify neutrophil migration into the liver during ALA, liver leukocytes isolated from the abscessed liver region (abscess), an unaffected (healthy) part of the liver, and from the livers of sham-operated mice (sham) were analyzed by flow cytometry. Neutrophils were identified as CD11b^+^, Ly6C^+^ and Ly6G^+^ cells [22] (Figure 2B). The proportion of neutrophils in the abscessed liver tissue was highest on Day 1 one and then decreased over time up to Day 5 post-infection. These results were consistent with the immunohistochemistry results. Additionally, there was a slight increase in the proportion of neutrophils in the non-abscessed part of the affected liver lobe that also decreased over time (Figure 2B).

To investigate the role of neutrophils in abscess formation, mice were subjected to immune depletion using anti-Ly6G and anti-GR1 (Ly6G^+^- and Ly6C^+^-reactive) antibodies [27] prior to intrahepatic amebic infection. Depletion of neutrophils (Ly6G^+^CD11b^+^) and Ly6C^+^ monocytes (Ly6C^hi^CD11b^+^) was confirmed by flow cytometry (Figure 2C). Depletion of Ly6G^+^CD11b^+^ cells was greatest one day after antibody treatment with either anti-GR1 (*P*<0.01) or anti-Ly6G (*P*<0.01) antibodies. Treatment with either antibody resulted in reduced numbers of Ly6G^+^CD11b^+^ cells up to six days post-depletion (Figure 2C, 1). Ly6C^hi^CD11b^+^ monocyte numbers were significantly reduced after a single treatment with anti-GR1 but not with Ly6G on Day 1, 2 and 4 post depletion. However, six days post depletion, the blood counts of Ly6C+ monocytes raised indicative for a reduction in anti-GR1 antibody level (Figure 2C, 2).

Two days after antibody depletion, mice were infected intra-hepatically with *E. histolytica* trophozoites. T_2_-weighted MRI spin echo analysis of infected livers enabled 3-dimensional analysis of liver lesions during the course of abscess development and quantification of abscess size (Figure 2D). There was a slight reduction in abscess size following neutrophil depletion using the anti-Ly6G antibody, but this result was not statistically significant. By contrast, depletion with anti-GR1 antibody resulted in a significant decrease in the size of the liver abscess as early as Day 3 post-infection (Figure 2E). Thus, Ly6C^+^ mononuclear cells, but not Ly6G^+^ neutrophils, appear to be critical cell mediators of tissue destruction during ALA formation.

### Role of macrophage subsets in ALA development

**Figure 3:**
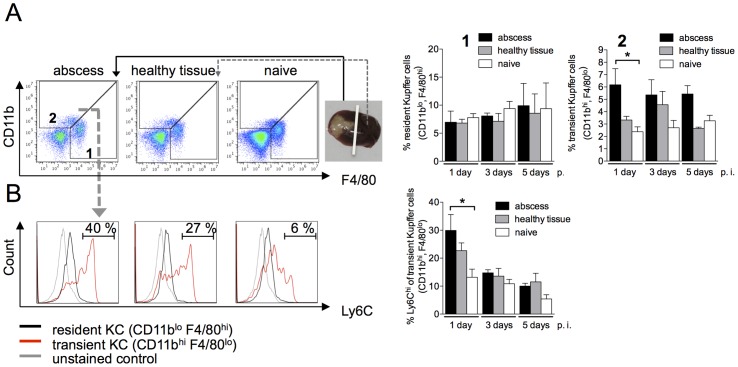
Characterization of Kupffer cell populations during ALA. (A) Gating strategy to define liver macrophage subpopulations in the abscessed region of an infected liver (abscess), a healthy region from the same liver lobe (healthy tissue), and liver tissue from a naïve animal (naïve) at the indicated time points post-infection. Resident Kupffer cells were defined as CD11b^lo^F4/80^hi^ cells (subset 1); transient inflammatory monocyte-derived Kupffer cells were defined as CD11b^hi^F4/80^lo^ (subset 2). (B) Representative histograms depict Ly6C expression levels. Data are shown in the bar graphs as mean ± SEM of two independent experiments at the indicated time points post infection (n = 6 animals/group); *P*-values were determined by the unpaired Student's t-test; ^*^ *P*<0.05.

To narrow down the cell subset involved in liver tissue damage from the heterogeneous population of mononuclear phagocytes, we monitored the recruitment of three distinct macrophage subsets to the site of the developing abscess. Mononuclear cell subsets were identified based on differential expression of the surface markers CD11b, F4/80, Ly6C and Ly6G [28]. Resident Kupffer cells in the liver were defined as CD11b^lo^F4/80^hi^Ly6G^−^ cells, whereas CD11b^hi^F4/80^lo^Ly6G^−^ macrophages represented a transient inflammatory stage from blood monocyte to tissue macrophage (transient Kupffer cells). This latter subpopulation was further differentiated based on expression of the monocyte marker Ly6C. Gradient-purified liver leukocytes were isolated from the abscess region, from an unaffected region of the same liver lobe, and from livers of naïve mice, and the mononuclear subpopulations were assessed by flow cytometry (Figure 3A).

Following infection with *E. histolytica*, the population of CD11b^lo^F4/80^hi^ macrophages, representing resident Kupffer cells, remained stable and did not differ between infected and naïve mice (Figure 3A, subset 1). By contrast, CD11b^hi^F4/80^lo^ cells, representing transient Kupffer cells, were more abundant in the abscess on Day 1 post-infection compared with adjacent healthy tissue or naïve liver tissue (*P*<0.05) (Figure 3A, subset 2). Of these, 30% also expressed a high level of Ly6C, indicating that they were derived from monocytes. During the course of ALA development, the subset of liver cells expressing high levels of Ly6C was lost, indicating the differentiation of monocytes into liver macrophages (Figure 3B).

### Role of resident Kupffer cells in ALA development

**Figure 4:**
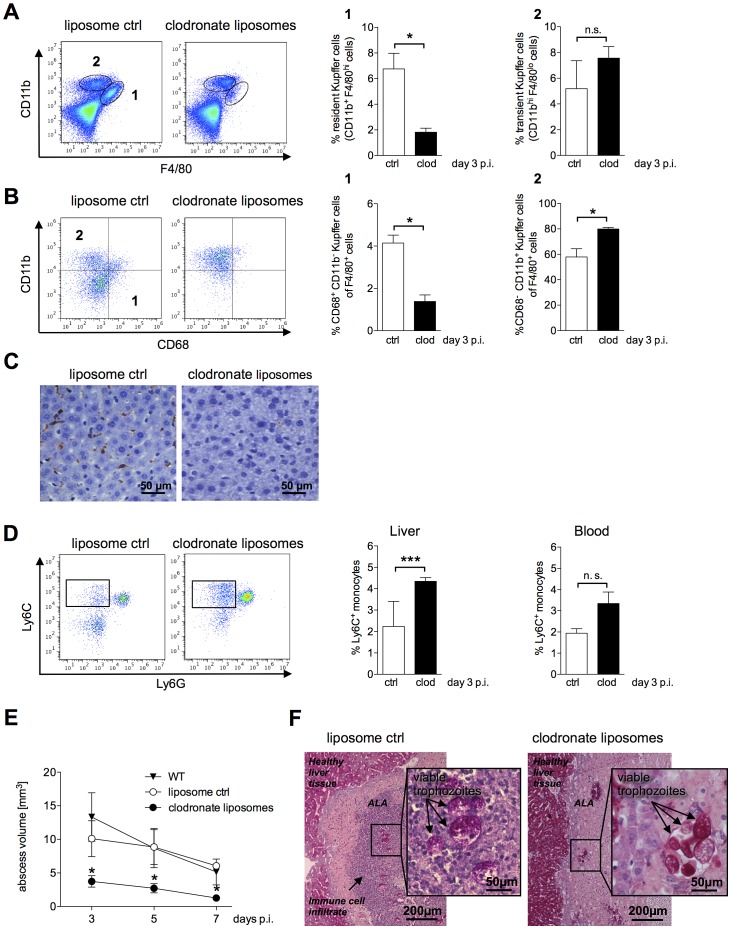
ALA formation following depletion of Kupffer cells by clodronate liposomes. (A) Gating strategy to define resident CD11b^+^F4/80^hi^ (subset 1) and transient inflammatory CD11b^hi^F4/80^lo^ (subset 2) Kupffer cells in the livers of mice five days after a single intravenous (i.v.) administration of clodronate liposomes (clod) or empty liposomes (ctrl) three days post-infection. Data represent the mean ± SEM of three independent experiments (n = 3 animals/group). (B) Gating strategy to define CD11b^+^CD68^+^ (region 1) and CD11b^+^CD68^−^ (region 2) Kupffer cells following treatment with clodronate liposomes (clod) or empty liposomes (ctrl) three days post-infection. (C) Immunohistochemical staining of liver tissue sections two days post i.v. administration of empty liposomes (ctrl) or clodronate liposomes (clod) using an anti-F4/80 antibody; Kupffer cells are indicated by the brown staining. (D) Gating strategy to define CD11b^+^Ly6G^−^Ly6C^+^ inflammatory monocytes derived from total liver and blood leukocytes five days post-clodronate treatment and three days post-infection. Data represent the mean ± SEM of three independent experiments (n = 3 animals/group). (E) Abscess size in wild-type (WT), clodronate-treated (clod), and control (ctrl) mice was monitored by MRI at the indicated times post-infection. Data represent the mean ± SEM of two experiments (3–4 mice/group). (F) PAS staining of abscessed liver tissue sections from control (ctrl) or clodronate-treated mice three days post-treatment and one day post-infection. Arrows indicate *E. histolytica* trophozoites. Data represent the mean ± SEM; *P*-values were determined by the Mann-Whitney U and unpaired Student's t test (^*^ *P*<0.05).

Resident Kupffer cells in the liver can exert hepatotoxic effects via expression of pro-inflammatory cytokines such as TNFα and IL-1β, as well as effector molecules such as NO [11]. To investigate the contribution of Kupffer cells to host tissue destruction during ALA, mice were subjected to cell depletion using clodronate [29]. Clodronate treatment significantly reduced the proportion of resident Kupffer cells (CD11b^+^F4/80^hi^) [28] in the liver (Figure 4A, subset 1). By contrast, transient, inflammatory monocyte-derived Kupffer cells (CD11b^hi^F4/80^lo^) were unaffected by clodronate treatment (Figure 4A, subset 2). Kinoshita et al. defined an “activated”, tissue destructive Kupffer cell population by the expression of CD68 (CD68^+^CD11b^−^F4/80^+^). We found that these cells were also diminished in the livers of infected clodronate-treated mice (Figure 4B, 1), whereas the proportion of non-activated (CD68^−^ CD11b^+^F4/80^+^) Kupffer cells increased (Figure 4B, 2) [15]. Similarly, immunohistochemical staining of liver sections from clodronate-treated mice revealed a complete loss of F4/80^+^ Kupffer cells (Figure 4C). Moreover, there was an increase in Ly6C-expressing, CD11b^+^Ly6G^−^ monocytes in the liver and blood of clodronate-treated mice compared with control animals (Figure 4D).

Neutrophils were unaffected by clodronate treatment; moreover, there was a significant increase in the relative numbers of these cells in the abscess and healthy liver tissue (data not shown).

ALA formation in infected mice was monitored by MRI from Day 3 to Day 7 post-infection. There was a significant reduction in abscess volume in clodronate-treated mice compared with that in control mice treated with empty liposomes or in untreated, infected wild-type mice (Figure 4E).

We also examined whether the viability of *E. histolytica* trophozoites, which have strong phagocytic potential, was affected by clodronate treatment. Based on PAS staining, trophozoite membranes appeared to be intact, and phagocytosis of erythrocytes was unaffected by clodronate treatment (Figure 4F). Interestingly, the massive influx of immune cells into the abscess was abolished by clodronate treatment (Figure 4F). In addition, re-isolation experiments indicated viable *E. histolytica* trophozoites in all of the animals treated with clodronate liposomes irrespectively whether ameba were isolated on Day 1 or Day 3 post-infection (data not shown).

### The role of Ly6C^+^ inflammatory monocytes in ALA progression

**Figure 5:**
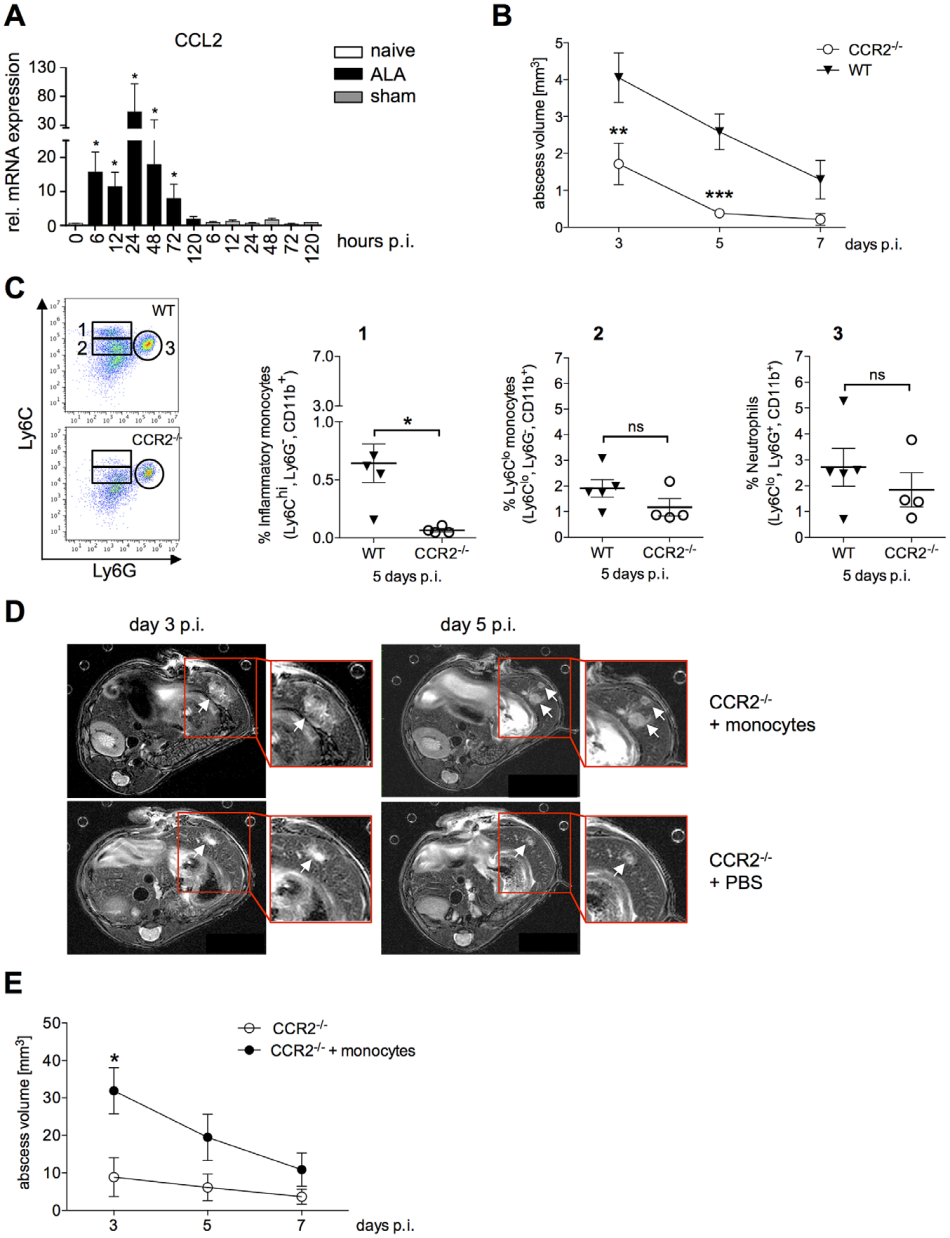
Role of Ly6C^hi^ inflammatory monocytes in abscess formation. (A) CCL2 mRNA levels in liver tissue of mice infected with *E. histolytica* trophozoites (ALA), sham-operated mice (sham) and naïve mice (naïve) at the indicated times (n = 4). (B) Abscess volume was determined by MRI at the indicated time points in wild-type (WT) and CCR2^−/−^ mice. Data represent the mean ± SEM of three independent experiments (n = 3–4 animals/group). (C) Gating strategy to define liver leukocytes from wild-type (WT) and CCR2^−/−^ mice five days post-infection. Inflammatory monocytes were defined as Ly6C^hi^Ly6G^−^CD11b^+^ cells (region 1); Ly6C^lo^ monocytes were defined as Ly6C^lo^Ly6G^−^CD11b^+^ cells (region 2); and neutrophils were defined as Ly6G^+^Ly6C^lo^CD11b^+^ cells. (D) Representative MRI images of abscesses (arrows) in infected wild-type (WT) and CCR2^−/−^ mice that received an adoptive transfer of CD115^+^ WT monocytes 6 hours post-infection; time post-infection is indicated. (E) MRI-based determination of abscess volume at the indicated times. Shown are representative data (mean ± SEM) of one out of two independent experiments (each 4–5 animals/group); *P*-values were determined by the Mann-Whitney U test (^*^ *P*<0.05; ^**^ *P*<0.01; ^***^ *P*<0.001).

Ly6C^hi^-expressing inflammatory monocytes are precursors of inflammatory tissue macrophages, the same cells that potentially mediate host tissue damage. Migration of Ly6C^hi^ monocytes from the bone marrow into the circulation is controlled by the expression of CCR2 and its cognate ligand, CCL2 [30]. Using qPCR, we found a significant increase (*P*<0.04) in CCL2 mRNA expression levels in the livers of infected mice compared with control mice as early as 6 h and up to 24 h post-infection (Figure 5A). Of note, in sham-operated animals (intrahepatic injection of culture medium), there was no increase in the expression of CCL2 mRNA.

To investigate the importance of Ly6C^hi^ monocyte recruitment in ALA, CCR2^−/−^ mice were infected with *E. histolytica* trophozoites, and the abscess volumes were determined by MRI. There was a significant reduction in abscess size compared with that in wild-type mice as early as three days post-infection (Figure 5B), with a further reduction seen at five days post-infection (*P*<0.0001). By contrast to wild-type mice, CCR2^−/−^ mice had almost fully recovered from the abscess lesions at seven days post-infection (Figure 5B). Analysis of liver leukocytes from infected mice revealed a significant decrease in Ly6C^hi^Ly6G^−^CD11b^+^ inflammatory monocytes in CCR2^−/−^ mice five days post-infection compared with wild-type mice (Figure 5C, 1). By contrast, the proportion of Ly6C^lo^Ly6G^−^CD11b^+^ monocytes, which are thought to be involved in wound healing and tissue repair, did not appear to be affected (Figure 5C, 2). Likewise, there was no difference in the neutrophil population between infected CCR2^−/−^ and wild-type mice (Figure 5C, 3).

To confirm the role of Ly6C^hi^ monocytes in promoting abscess development, adoptive transfer of purified, bone marrow-derived CD115^+^ monocytes was performed in CCR2^−/−^ mice 6 h after intrahepatic amebic infection. As shown by MRI, abscess formation was more diffuse and multifocal in CCR2^−/−^ mice following adoptive transfer of monocytes compared with the focal and dense character of the abscess formed in a wild-type mouse (Figure 5D). Importantly, there was an increase in abscess volume (*P*<0.05) at Day 3 post-infection in animals that were nearly ALA-resistant prior to transfer (Figure 5E). These results indicated that Ly6C^hi^ inflammatory monocytes contribute substantially to liver tissue destruction during ALA development.

### Influence of NO and TNFα on ALA formation

**Figure 6:**
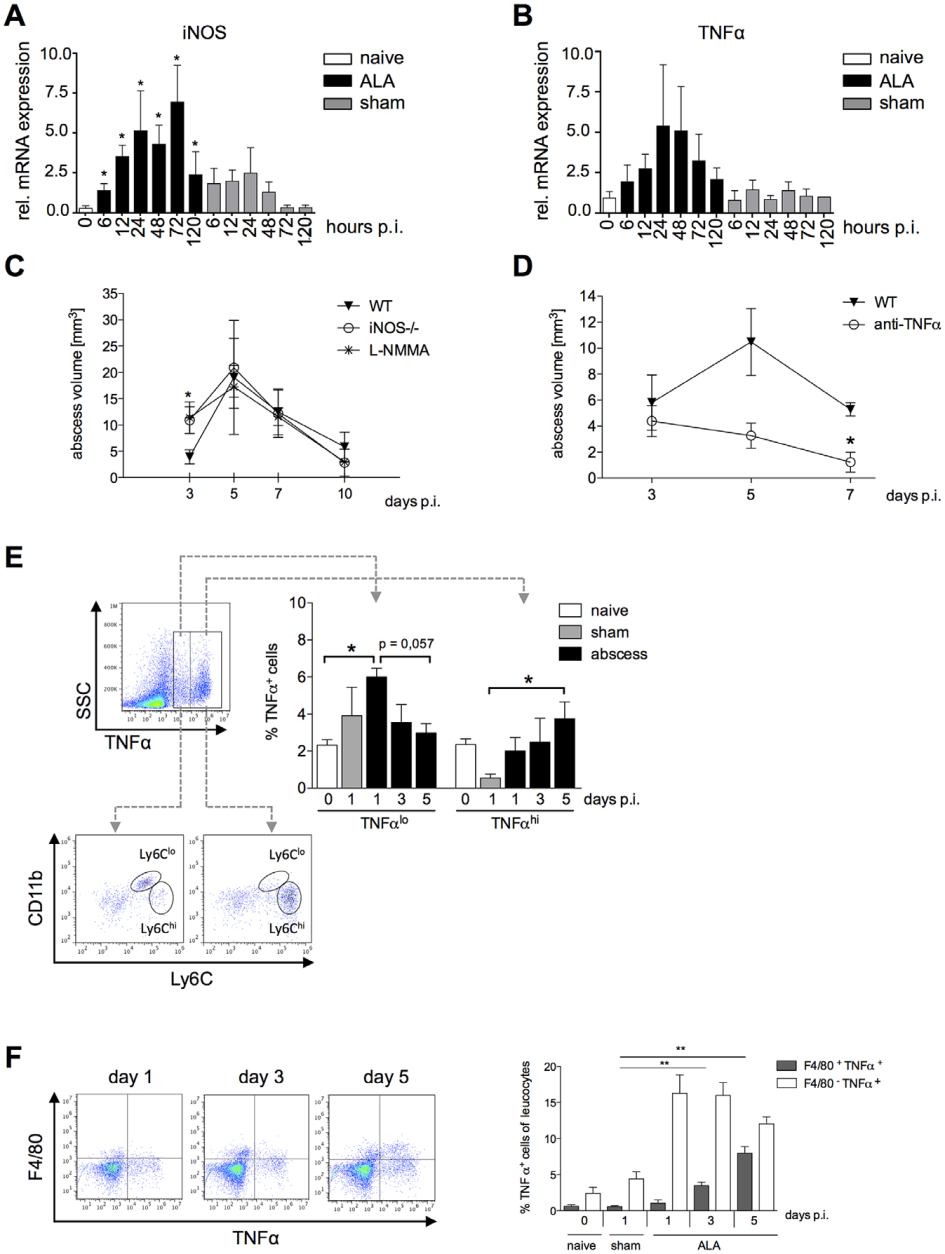
Contribution of NO and TNFα to liver tissue damage during ALA. iNOS (A) and TNFα (B) mRNA levels in liver tissue of mice infected with *E. histolytica* trophozoites (ALA), sham-operated (sham) and naïve (naïve) mice at the indicated times post-infection (n = 4). (C and D) MRI-based determination of abscess volume in wild-type (WT), iNOS^−/−^ as well as L-NMMA treated mice (C) and in wild-type mice treated with an anti-TNFα antibody 24 h before infection (D). Time post-infection is indicated (4–5 animals/group). (E) Gating strategy to define liver leukocytes producing TNFα; cells were defined as low (TNFα^lo^) and high (TNFα^hi^) producers of TNFα. Leukocytes were isolated from infected (ALA), sham-operated (sham) and naïve (naïve) mice at the indicated times post-infection. Cells were further characterized according to the expression of Ly6C as CD11b^+^Ly6^lo^ and CD11b^+^Ly6C^hi^ monocytes. (F) Gating strategy to define the numbers of TNFα-positive F4/80^+^ Kupffer cells in livers of naive, sham immunized and infected mice on indicated time points. Data represent the mean ± SEM of two independent experiments (2–5 animals/group); *P*-values were determined by the Mann-Whitney U test (^*^ *P*<0.05, ^**^ *P*<0.01, ^***^ *P*<0.001).

NO, produced by inducible nitric oxide synthase (iNOS), and TNFα are mediators of monocyte and macrophage cytotoxicity in host tissues [31]. Using qPCR, we investigated changes in iNOS and TNFα mRNA expression levels following intrahepatic amebic infection. Expression of iNOS and TNFα mRNA was upregulated following infection compared with that in naïve or sham-operated mice for up to 24 h, and declined thereafter (Figure 6A and B).

To determine the relative contributions of iNOS and TNFα to tissue destruction following amebic infection, we induced ALA in iNOS^−/−^ mice, in mice treated with the NO-inhibitor L-NMMA and in TNFα-neutralized mice, respectively (Figure 6C and D). In iNOS-deficient or L-NMMA treated mice, which lack NO, differences in abscess formation were evident only up to three days post-infection (Figure 6C), whereas in mice lacking TNFα, abscess sizes were reduced relative to wild-type mice on Day 5, and mice had largely recovered by seven days post-infection (*P*<0.05) (Figure 6D). These results indicated that TNFα plays a critical role in promoting disease progression during ALA.

To identify a potential source of TNFα during ALA, leukocytes isolated from the livers of infected, naïve and sham-operated mice were stimulated *ex vivo* with heat-killed listeria lysate and then analyzed by flow cytometry (Figure 6E). We identified a cell population that produced a low level of TNFα (TNF^lo^) and a population that produced a high level of TNFα (TNFα^hi^). At one day post-infection, there were significantly more TNF^lo^-expressing cells in infected mice compared with healthy, naïve mice (*P*<0.05). The proportion of these cells gradually decreased until Day 5 post-infection. By contrast, the proportion of TNFα^hi^-expressing cells increased during the course of ALA development. Further characterization of TNFα-producing cells based on Ly6C expression revealed that TNFα^lo^-expressing cells comprised equal proportions of Ly6C^lo^- and Ly6C^hi^-expressing monocytes, while TNFα^hi^-expressing cells comprised mainly Ly6C^hi^ inflammatory monocytes. In addition, we estimated the proportion of TNFα^+^F4/80^+^ Kupffer cells during ALA. Interestingly, the proportion of TNFα-producing Kupffer cells was low at the onset of intrahepatic amebic infection but raised until Day 5, correlating with the increasing numbers of Ly6C^hi^ TNFα^hi^ monocytes during ALA (Figure 6F).

Thus, mainly Ly6C^hi^ inflammatory monocytes promote disease progression during *E. histolytica* infection and mediate liver tissue damage, in part, through elevated expression of TNFα.

## Discussion

*E. histolytica* is a protozoan parasite that normally persists as a harmless commensal organism in the intestine of humans. Parasite pathogenicity factors identified and characterized to date have been implicated in survival within the gut by mediating attachment to colonic mucins, as well as the uptake, killing and digestion of bacteria engulfed from the gut flora [32]. However, these effector molecules also enable penetration of the parasite into the submucosa, leading to chronic ulcerative gut inflammation. During this process, the parasite can spread via the blood stream to other organs of the body, in particular in the non-permissive microenvironment of the liver.

Of long-standing debate is whether parasite effector molecules or host factors are responsible for the tissue destruction observed during ALA.

To investigate whether host immune mechanisms contribute to ALA development, a recently established mouse model for ALA was used. In contrast to other rodent models for ALA, only the mouse model allows state of the art immunological investigations.

Other immunocompetent animals used as models for ALA include the highly susceptible hamster and the gerbil (*Meriones unguiculatus*) [7], [33]–[35].

Like in the gerbil, but in contrast to the hamster model, the time course of abscess formation in the mouse model is self-limited and amebic lesions are cleared within 30 days post infection. However, similar to abscess formation in hamsters and gerbils, the mouse model shows a massive infiltration of immune cells at the site of infection, followed by necrosis in the center of the abscess at a later time point [9]. Epitheloid cells, indicative for granuloma formation that is usually detected in the infected liver of the hamster or the gerbil model for ALA [34], are not characteristic for ALA formation in the mouse. However, as is also shown in the hamster, amebic trophozoites are rarely detected in direct contact to hepatocytes leading to the assumption that tissue destruction is a result of accumulation and subsequent lysis of leukocytes and macrophages, as already suggested by others [7].

In agreement with histological studies in other animal models for ALA, neutrophils were the first immune cells to infiltrate the liver during the acute phase of invasive amebic infection. Neutrophils are thought to exert a protective role during ALA [36], and their presence at the site of infection was consistent with previous studies showing that *E. histolytica*-derived surface peptides act as neutrophil chemoattractants [37]. More recently, classical danger signals and chemokines released from injured hepatic cells were shown to be involved in the recruitment of neutrophils as well [10].

Using immunohistochemistry and quantitative flow cytometry, we showed that neutrophils comprised the majority of infiltrating immune cells in the abscess one day after intrahepatic amebic infection, and localized close to amebic trophozoites. By Day 3 post-infection, when the abscess reached its maximum size, neutrophil staining was more diffuse, suggesting that substantial cell death was occurring. By seven days post-infection, neutrophils represented a minor population of immune cells in the abscess, suggesting that most neutrophils had already undergone cell death.

Neutrophils play a central role in host defense against invasive microorganisms, and *in vitro* stimulation with cytokines (i.e. IFNγ and TNFα) or LPS triggers amebicidal activity, presumably by inducing expression of reactive oxygen species (ROS) [36]. However, ROS, as well as the diverse array of proteases derived from neutrophils and expressed during the respiratory burst, can also mediate host tissue damage. This event is not necessarily detrimental to the host, since it can also lead to the initiation of wound healing [38]. To investigate the contribution of neutrophils to liver tissue destruction during ALA, we performed immune depletion experiments using anti-Ly6G and anti-GR1 monoclonal antibodies (mAbs). Anti-Ly6G recognizes the neutrophil-specific cell surface molecule Ly6G, and selectively depletes neutrophils. By contrast, anti-GR1, which is a classical neutrophil depletion agent, also recognizes Ly6C-expressing monocytes [39]. Immune depletion experiments in severe combined immune deficient (SCID) mice using anti-GR1 mAbs demonstrated a protective role for neutrophils during ALA. Abscesses in immune depleted mice were significantly larger, contained fewer immune cells, and had a greater number of amebic trophozoites compared to. However, SCID mice are not able to mount an appropriate immune response because they lack T and B lymphocytes; therefore, neutrophils play a more prominent role in the ALA SCID mouse model that may not reflect a normal physiological setting [40].

Interestingly, compared with the data obtained from SCID mice, the current results showed nearly the opposite phenomenon. Despite the fact that immune depletion of neutrophils with anti-Ly6G mAbs led to a significant decrease in the number of neutrophils, liver abscess size was slightly smaller compared with that in wild-type mice. Thus, our results indicated that neutrophils do not have a beneficial role in ALA and, in fact, contribute to liver damage during amebic infection. Of note, concomitant depletion of Ly6C-expressing monocytes using anti-GR1 mAb led to an even more pronounced reduction in abscess volume, which indicates that Ly6C^+^ inflammatory monocytes, as precursors of inflammatory F4/80-expressing macrophages [11], are also involved in liver tissue destruction during hepatic amebiasis.

In contrast to neutrophils, on Day 1 post-infection, F4/80^+^ macrophages appeared to be less abundant and were not in direct contact with amebic trophozoites. At Day 5 post-infection, these cells formed a margin around the center of the abscess and eventually infiltrated the abscess completely. Using flow cytometry to further differentiate the F4/80^+^ macrophage subsets involved in ALA, we found no differences over the course of ALA in the number of resident Kupffer cells (CD11b^lo^F4/80^hi^); rather, there was a strong increase in transient inflammatory liver macrophages (CD11b^hi^F4/80^lo^) in the abscessed liver area. On Day 1 post-infection, the majority of these cells also expressed the monocyte surface marker Ly6C; however, over time, Ly6C expression was lost, suggesting that these cells originated as infiltrating inflammatory Ly6C^hi^ monocytes.

Resident Kupffer cells are the first macrophage population in the liver to come into contact with invading *E. histolytica* trophozoites. *In vitro* and *in vivo* studies support a critical role for these cells in killing and eliminating parasites. Activated by pro-inflammatory cytokines or colony stimulating factor-1, resident Kupffer cells produce NO, which is amebicidal, as well as ROS, perhaps the most effective molecule for amebic killing [36], [41].

Activated Kupffer cells also contribute to liver tissue destruction in models of viral-induced or hepatotoxic liver diseases [20], [42]–[44]. In these models, activated Kupffer cells express CD68 and exert hepatotoxic effects by secreting inflammatory mediators such as TNFα, Fas ligand, or ROS [19], or by promoting the accumulation of cytotoxic T cells in the liver [45]. In the current study, the depletion of Kupffer cells by gadolinium chloride (GdCl_3_) or clodronate liposome treatment almost completely abolished ALA pathology. Surprisingly, Kupffer cells also contributed substantially to liver damage during ALA formation. The number of abscesses in clodronate-treated mice was significantly reduced compared with control mice. Using the gating strategies described by Karlmark et *al.* [28] and Kinoshita et *al.* [15], we demonstrated a substantial reduction in F4/80^hi^CD11b^+^ cells, as well as F4/80^+^CD68^+^ Kupffer cells, in the livers of clodronate-treated animals. Thus, activated CD68^+^ Kupffer cells play a major role in the immune pathology observed during ALA. Although abscess formation was significantly reduced, amebic trophozoites within the remaining lesions appeared healthy as determined by histology. Trophozoites were still engaged in phagocytosis of host cells and exhibited strong PAS staining, indicative of intact cell membranes. Interestingly, we found a near-complete absence of immune cells in the residual abscess lesions of clodronate-treated mice, which indicates that Kupffer cells may be involved in the initiation of inflammation during abscess formation. The high re-isolation rate of viable ameba trophozoites from the liver up to Day 5 post clodronate treatment further indicates a minor direct role of *E. histolytica* for liver damage.

CD11b^+^Ly6C^+^ blood monocytes were recruited to the liver at an early time point after amebic infection. qPCR analysis indicated that the mRNA expression level of CCL2, one of the most potent chemoattractants of inflammatory Ly6C^hi^ monocytes, was upregulated within hours after intrahepatic infection with *E. histolytica*. CCL-2 binds to CCR2-expressing Ly6C^+^ monocytes and initiates the migration of Ly6C^hi^ monocytes from the bone marrow into the circulation. Knockout mice lacking CCR2 often show an increased susceptibility to microbial infections [21].

Abscess formation was almost abolished in CCR2^−/−^ mice. This was accompanied by a significant reduction in the percentage of CD11b^+^Ly6C^hi^ inflammatory monocytes, whereas the percentage of CD11b^+^Ly6C^lo^ monocytes and neutrophils was unchanged or even elevated. Adoptive transfer of wild-type CD115^+^ monocytes into CCR2^−/−^ mice restored abscess formation, and transferred monocytes were confirmed as mainly Ly6C^hi^-expressing cells. Thus, CCR2^+^Ly6C^hi^ inflammatory monocytes appear to play a critical role in abscess formation. Interestingly, the abscesses in these mice appeared multifocal, in contrast to the dense appearance of the liver lesions in wild-type mice. These findings were similar to those seen with acetaminophen-, carbon tetrachloride-, or diet-induced models of liver injury [24]–[26], [46]. However, we do not believe that ALA is a “toxic-like” type of liver destruction in response to the complex culture medium co-injected with the amebic trophozoites. In contrast to other effector molecules, such as TNFα or iNOS, CCL2 mRNA expression was upregulated only in the presence of amebic trophozoites.

Nitric oxide (NO) is reported to be a major cytotoxic molecule produced by macrophages that inhibits amebic pathogenicity factors like cysteine proteinases and alcohol dehydrogenase 2 [41]. In addition, *in vitro* data suggested that *E. histolytica* trophozoites or amebic components might modulate macrophages functions i.e. NO production [47] by competing for the substrate L-arginine [41]. Seydel et *al.* have shown that mice, lacking both the IFNγ receptor and iNOS (129/Sv/Ev 3 C57BL/6 (iNOS1/2)) were unable to control ALA [48]. In contrast to the current opinion, our results indicate a minor contribution of NO for ALA control. Both iNOS^−/−^ and L-NMMA treated mice indicated only moderate effect of NO on ALA development within the first three days after intrahepatic infection and argues that the effect seen in the double knock-out mice used by Seydel et *al.* might primarily be due to the lack of the ability to activate immune cells via IFNγ, a cytokine that is crucial in the control of ALA [9], [36], [49]. TNFα is a key cytokine that correlates with macrophage dependent tissue destruction [11]. TNFα mRNA expression was induced at the onset of ALA, but this was also observed, albeit to a lesser extent, in sham-operated mice. In contrast, intracellular production of TNFα protein in re-stimulated liver leukocytes was higher in *ex vivo* cultures from infected mice compared with sham mice, which suggests that TNFα was secreted specifically in response to *E. histolytica* infection. Ly6C^hi^ inflammatory monocytes produced high levels of TNFα, whereas Ly6C^lo^ monocytes expressed lower levels of TNFα. In addition, we found low numbers of TNFα-producing Kupffer cells that increased significantly during the disease progression. Neutralization of TNFN during amebic infection resulted in a decrease in the size of abscesses, supporting a critical role of this cytokine in liver tissue destruction. However, further experiments are required to investigate the crosstalk between Kupffer cells and monocytes leading to tissue destruction during ALA.

In conclusion, data from the current study demonstrated that host immune responses play a major role in the liver pathology induced by *E. histolytica* infection. Challenging previous assumptions, we found that the contribution of neutrophils to ALA may be overestimated in certain models, since they neither contributed substantially to tissue destruction nor the progression of ALA. Rather, Kupffer cells and inflammatory monocytes are likely the main cell populations responsible for tissue destruction. TNFα was a critical cytokine mediator of tissue destruction during ALA. Additional studies are needed to unravel the complex interplay between activated Kupffer cells and inflammatory monocytes during ALA development.

## Methods

### Ethics statement

The study was carried out in accordance with the guidelines from the German National Board for Laboratory Animals and approved by the Authority for Consumer Protection and Health, Hamburg, Germany (ethical permits 23/09, 41/11).

### Mice

Male C57BL/6 mice (aged 10 to 12 weeks) were obtained from Charles River Laboratories (Sulzfeld, Germany); CCR2^−/−^ mice were kindly provided by Daniel Engels (University Clinic of Bonn, Germany); iNOS^−/−^ mice (Max-Plank Institute for Infection Biology, Berlin, Germany) were housed and bred in the animal facility of the Bernhard Nocht Institute for Tropical Medicine, Hamburg, Germany. Mouse strains were backcrossed for more than 10 generations against a C57BL/6 background.

### Cultivation of *E. histolytica*

ALA was induced using virulent cell line B derived from *E. histolytica* HM-1:IMSS through long-term culture [50]. Trophozoites of HM-1:IMSS were grown in axenic cultures in TYI-S-3 medium [51].

### Induction of ALA and monitoring of liver abscess development using MRI

ALA was induced by intrahepatic injection [52] of 5×10^4^ virulent *E. histolytica* trophozoites, cell line B [50], [52] as described previously [9]. Sham-operated mice received TYI-S-3 medium alone. MRI was performed at the indicated times post-infection using a small animal 7 tesla MRI scanner (ClinScan, Bruker Biospin GmbH, Ettlingen, Germany). MRI was performed using a T_2_-weighted turbo spin echo sequence (T_2_TSE). Total abscess volume was calculated by measuring the region of interest (ROI) in each slice showing the abscess on transversal sections of the abdomen using OsiriX Imaging Software DICOM Viewer (Open-source version 32-bit 4.1.1).

### Histology and immunohistochemistry

Liver tissue from ALA mice was fixed in formalin (4%) and then embedded in paraffin. Sections (0.2 µm) were stained with H&E, PAS, or prepared for immunohistochemistry. Neutrophils were visualized using rabbit anti-mouse 7/4 antibody (clone 7/4; Cedarlane; 1∶800 dilution) and macrophages were visualized using rat anti-mouse F4/80 antibody (clone Cl:A3-1; Serotec; 1∶3000 dilution) using standard methodology. Antibodies were detected using DCS SuperVision Single Species horse-radish peroxidase (HRP)-Polymere (Innovative Diagnostic-Systems) and the samples were counterstained with hemalaun.

### Immune depletion and neutralization of TNFα

Immune depletion of neutrophils was performed by intraperitoneal (i.p.) administration of anti-Ly6G mAb (clone 1A8, BioXcell; 500 µg/animal) on Days -2, -1, and 0 (relative to the day of infection on Day 0), and on Days 1 and 2 post-infection. Similarly, immune depletion with GR-1 mAb (clone RB6-8C; 300 µg/animal) was performed by i.p. administration on Day -2 and Day 1 post-infection. To neutralize TNFα, rat anti-TNFα mAb (V1qH8, Abcam; 500 µg/animal) was administered i.p. 24 h prior to intrahepatic infection. Rat IgG (Jackson Laboratories Inc; 300 µg/animal) was used as a control mAb and administered i.p. as described for depletion mAbs.

### Adoptive transfer of bone marrow monocytes

Cell suspensions were prepared from the bone marrow of C57BL/6 mice. Monocytes were labeled with biotinylated anti-CD115 mAb (clone AFS98; eBioscience) and then purified using streptavidin MicroBeads (Miltenyi) and magnetic-assisted cell sorting. Adoptive transfer was carried out using 1×10^6^ monocytes via lateral tail vein injection 6 h post-infection.

### Functional inhibition of macrophages by clodronate and L-NG-monomethyl Arginine citrate (L-NMMA) treatment

Mice were injected intravenously in the tail vein using 200 µl Clodronate liposome solution (ClodronateLiposomes.org, Amsterdam, Netherland) or empty liposomes as control two days prior to intrahepatic infection with *E. histolytica* trophozoites.

L-NMMA (2 mg/in 100 µl phosphate buffered saline/animal) was applied i.p daily from Day 2 before until Day 7 after amebic challenge.

### Flow cytometry

Leukocytes were isolated from liver and whole blood. Livers were perfused with ice-cold PBS, minced, and then filtered through a 70 µm nylon mesh. After washing, the cell pellet was subjected to density gradient centrifugation using 30% Nycodenz (Nycoprep, Universal). Leukocytes were isolated from the interface and subjected to red blood lysis (RBL). Fc-**γ** receptors were blocked with rat anti-mouse CD16/CD32 antibody (Fc-**γ** III/II receptor) and then cells were stained with the indicated antibodies for FACS analysis.

Whole blood was subjected to RBL, blocked as described above and then stained with the indicated combinations of the following mAbs: CD11b (cl: M1/70); CD115 (cl: AFS98); F4/80 (cl: BM8), GR1 (cl: RB6-8C5), CD68 (cl: FA-11), Ly6G (cl: 1A8), Ly6C (cl: HK1.4), Isotype IgG1κ (BioLegend). Data were acquired with a BD Accuri C6 Flow Cytometer (Accuri Cytometers Inc.) and analyzed with FlowJo 7.6.3 (Treestar).

### Intracellular staining of TNFα

For intracellular TNFα staining, purified spleen and liver lymphocytes (1×10^6^ cells) were stimulated with 10 µl of heat killed *Listeria monocytogenes* (1.6×10^9^ HKL/ml) [31]. Un-stimulated control cells were incubated with 1 ml of complete RPMI 1640 medium. Liver and spleen lymphocytes were stimulated for 30 min at 37°C in a 5% CO_2_ atmosphere and then incubated for additional 4 hours with Brefeldin A. After blocking in Fc-**γ** receptor blocking solution, cells were washed and then subjected to surface antigen staining using the antibodies described for flow cytometry. Following fixation (Becton Dickinson), cells were permeabilized in Perm/wash solution (1∶10 dilution; Becton Dickinson). Intracellular cytokine staining was performed using an anti-TNFα mAb (cl: MP6-XT22) and followed by FACS analysis.

### qPCR

For isolation of total RNA, abscessed liver material in an appropriate volume of Trizol (Ambion) was homogenized and subjected to isopropanol precipitation. Purification was performed using the RNeasy Mini Kit (Qiagen). cDNA was synthesized using the MaximaFirst Strand cDNA synthesis kit (Fermentas). qPCR was performed on a Rotor-Gene RG-3000 (Corbett Research) system using the Maxima SYBR Green qPCR Master Mix (Fermentas). Expression levels were calculated using the 2^−ΔΔCt^ method [53], normalized to ribosomal protein S9 (RPS9) and calibrated against expression measured at 120 hours in sham-operated mice. Calculations were performed using rotor-gene 6 Version 6.1 CR software (Corbett Research).

The following mouse specific primer sequences were used for amplification: iNOS *s*: TGGTGGTGACAAGCACATTT; iNOS *as*: TGGTGGTGACAAGCACATTT; TNFα *s*: AGTTCCCAAATGGCCTCCCTCTCA; TNFα *as*: GTGGTTTGCTACGACGTGGGCT; CCL3 *s*: ATGAAGGTCTCCACCACTGC; CCL3 *as*: GATGAATTGGCGTGGAATCT; CCL2 *s*: TCTCTCTTCCTCCACCACCA; CCL2 *as*: CGTTAACTGCATCTGGCTGA RPS9 *s*: CCGCCTTGTCTCTCTTTGTC; RPS9 *as*: CCGCCTTGTCTCTCTTTGTC

### Statistical analysis

The non-parametric Mann-Whitney U test and unpaired Student's t test were performed using Prism statistical software (GraphPad Prism 5).
